# A Hyperspectral Image Classification Approach Based on Feature Fusion and Multi-Layered Gradient Boosting Decision Trees

**DOI:** 10.3390/e23010020

**Published:** 2020-12-25

**Authors:** Shenyuan Xu, Size Liu, Hua Wang, Wenjie Chen, Fan Zhang, Zhu Xiao

**Affiliations:** 1 State Key Laboratory of Geo-Information Engineering, Xi’an 710054, China; syxu@hnu.edu.cn (S.X.); sklgie@163.com (H.W.); zhangfan01@stu.xidian.edu.cn (F.Z.); 2 College of Computer Science and Electronic Engineering, Hunan University, Changsha 410082, China; zhxiao@hnu.edu.cn; 3 College of Communication Engineering, Xidian University, Xi’an 710071, China; liusize@stu.xidian.edu.cn; 4 Business College, Central South University of Forestry and Technology, Changsha 410004, China

**Keywords:** hyperspectral image, multi-layered gradient boosting decision trees (mGBDTs), feature fusion, image classification

## Abstract

At present, many Deep Neural Network (DNN) methods have been widely used for hyperspectral image classification. Promising classification results have been obtained by utilizing such models. However, due to the complexity and depth of the model, increasing the number of model parameters may lead to an overfitting of the model, especially when training data are insufficient. As the performance of the model mainly depends on sufficient data and a large network with reasonably optimized hyperparameters, using DNNs for classification requires better hardware conditions and sufficient training time. This paper proposes a feature fusion and multi-layered gradient boosting decision tree model (FF-DT) for hyperspectral image classification. First, we fuse extended morphology profiles (EMPs), linear multi-scale spatial characteristics, and nonlinear multi-scale spatial characteristics as final features to extract both special and spectral features. Furthermore, a multi-layered gradient boosting decision tree model is constructed for classification. We conduct experiments based on three datasets, which in this paper are referred to as the Pavia University, Indiana Pines, and Salinas datasets. It is shown that the proposed FF-DT achieves better performance in classification accuracy, training conditions, and time consumption than other current classical hyperspectral image classification methods.

## 1. Introduction

Hyperspectral image (HSI) processing is one of the core research areas in the field of HSI remote sensing [1,2,3,4,5], which is an important means of earth observation. HSIs have many spectral bands and large amounts of data, many of which are redundant. Designing an algorithm and obtaining efficient performance is key in this field. There are two important points in HSI classification: proper feature representation and an efficient classification model.

Extended shape contours are constructed by mathematical operations of structural elements to form shapes of various sizes [6,7], and morphological attribute profiles obtained through more complex morphological operations [8,9,10] are representative in spatial information. In [11], a spline wavelet, which has the characteristic of spatial translation invariance, was used to extract spatial spectrum features. In order to improve the performance, multiple extension methods of SVM applied to the HSI field have been proposed [12]. Convolutional neural networks use local receptive fields to extract spatial information in images and local sharing mechanisms to reduce network training parameters. Convolutional neural networks are now being applied to HSI classification tasks with greater frequency [13,14,15,16]. In [17], the authors used a CNN in the study of depth representation methods based on spectral features, and the classification effect was better than the traditional SVM algorithm. In [18], the authors utilized CNN and obtained a higher accuracy in classification capabilities by learning pixel-pair features. In [19], the authors proposed a multi-region CNN classification method for HSIs.

However, in order to obtain better accuracy by using DNN models, much effort is needed to adjust the parameters of deep model. Furthermore, when the data change, the deep network also needs to adjust the network structure. Beyond that, there are problems caused by a vanishing gradient and an exploding gradient when using DNN for classification: overfitting and underfitting. Though a large amount of HSI data has been obtained due to advanced HSI remote sensing technology, there are only a few labeled HSI data. Without sufficient training data, it is difficult to obtain a satisfactory result for a DNN model with many parameters, due to intensive computing conditions. An efficient classification network is needed that can be applied to small devices, such as drones and remote sensing platforms.

Additionally, in HSI classification tasks, preprocessing-based methods and hybrid methods often use extracted features and classifiers to implement classification tasks. Therefore, the quality of the extracted features and the selected classifier obviously affects classification results. Feature extraction is used to transform original features into features with actual physical meaning, such as texture features, geometric features, and edge features in the image. Choosing appropriate features can reduce redundant information in the data and mine the potential deep-level information of the data, which will greatly help the subsequent classification.

Summarizing, the HSI classification problem is facing several technical challenges:

(i) How can the right features be chosen where multiple features can be fused?
(ii) For classification, how can model training be effective with a few parameters and low computational complexity?
(iii) How can a satisfactory classification model be obtained in a short time under limited hardware conditions?

To solve the above-mentioned problems, we strive to propose a new strategy via leveraging feature fusion and a multi-level gradient boosting decision tree (mGBDT) method [20]. The main contributions are outlined as follows.

- We extract extended morphology profiles, linear multi-scale spatial characteristics, and nonlinear multi-scale spatial characteristics as final features. The original data of the HSI is a three-dimensional image, and the spatial dependence complementary to the spectral information behavior is naturally another information source. The introduction of spatial information improves the possibility of pixel-by-pixel classification.
- We utilize a decision tree-based model, namely, mGBDT, which has fewer parameters and is easier to train. Compared with deep learning model, the proposed model is easy for theoretical analysis and practical training, and only requires simple hardware conditions to perform model training.

In this paper, feature fusion and a mGDBT model (FF-DT) for HSI classification is proposed. In Section 2, we briefly introduce works relevant to the proposed method. In Section 3, the structure of our method is presented. In Section 4, the experiment design is detailed. Section 5 shows the results. Finally, a summary of the paper is given.

## 2. Related Work

### 2.1. Principal Component Analysis

In [21], principal component analysis of a data matrix was proposed to extract the dominant patterns in a matrix in terms of a complementary set of score and loading plots, alleviating computing pressure and accelerating computing speed.

In [22], a folded PCA (F-PCA), in which both global and local structures were taken into account, preserved all useful properties of PCA. The work simplified the analysis of the high dimensional nature of a hyperspectral image.

With the improvement of sensor technology, the dimensions of hyperspectral images are generally in the tens to hundreds of dimensions. The amount of data is large and redundant. If it is trained, it takes a long time and requires good hardware. Thus, our purpose is to reduce the dimensionality of n-dimensional data to k-dimensional data, that is, to find a k-dimensional plane to represent this dataset. In order to increase the training rate, a certain amount of data needs to be reduced. In this paper, we use principal component analysis (PCA) to reduce the spectral dimension of the hyperspectral image.

### 2.2. Extended Morphological Features

In [23], a semisupervised classification algorithm for hyperspectral remote sensing images based on spectral and spatial information was proposed. The spatial information was extracted by building extended morphological profiles (EMPs) based on principle components of the hyperspectral image.

In [24], morphological profiles were used to fuse spectral and spatial information to produce improved classification results. Opening and closing morphological transforms were used in order to isolate bright (opening) and dark (closing) structures in images, where bright/dark means brighter/darker than the surrounding features in the images.

The morphological feature is a nonlinear method. The basic operations included are opening operation, corrosion, expansion, and closing operation. Given a set *A*, the structural element B corrodes *A*, and the output result is the set of the origin position of *B* when *B* is scanned by *A* and *B*.

(1) $$A⊙B=\left\{z|\left(B_{z}\subseteq A\right)\right\}$$

The expansion is to use the structural element *B* to scan from the beginning to touch the set *A* to find the set of the origin of *B*, so at least one element of *A* and *B* overlaps [25].

(2) $$\left.A⊕B=\left\{z|{\left[\hat{B}\right)}_{z}\cap A\right]\neq \emptyset \right\}$$

The opening and closing operations are defined by the sequence of corrosion and expansion. Corrosion before expansion is an opening operation, which can eliminate smaller bright spots and retain the characteristics of a larger bright area [26]. Expansion after corrosion is a closing operation, which can bridge narrower discontinuities and remove smaller dark spots in the image.

(3) $$MP^{\left(n\right)}\left(I\right)=\left[\varphi_{r}^{\left(n\right)}\left(I\right),\cdots ,\varphi_{r}^{\left(1\right)}\left(I\right),I,\cdots ,\gamma_{r}^{\left(n\right)}\left(I\right)\right]$$

where *n* is the number of open and closed operations, *I* is the image to be processed, $\varphi_{r}\left(I\right)$ is the closing operation, and $\gamma_{r}\left(I\right)$ is the opening operation. Because hyperspectral images have a large number of spectral dimensions, in order to utilize these spectral dimensions, extended morphological feature extraction methods are used first by reducing the spectral dimension of the hyperspectral image, then by extracting m principal components, and then extracting the morphological features of each component [27].

(4) $$EMP_{m}^{\left(n\right)}\left(I\right)=\left[MP_{1}^{\left(n\right)}\left(I\right),\cdots MP_{m}^{\left(n\right)}\left(I\right)\right]$$

## 3. Methodologies

Because the traditional method does not combine spatial information of hyperspectral imagery, we propose a hyperspectral image classification method based on feature fusion and an mGBDT. The method first uses PCA to reduce the original hyperspectral data, and then extracts its extensible morphological features (EMP), linear multi-scale features, and nonlinear multi-scale features from the reduced-dimensional data. The fusion of these three features is used as the input of the deep forest classifier. The experimental results prove that the hyperspectral classification method based on feature fusion and deep forest is efficient and has excellent performance, even on small samples.

### 3.1. Linear Multi-Scale Spatial Characteristics

The scale space aims to obtain images at different scales. In [28], the linear and nonlinear multiscale spatial characteristics are used in the proposed algorithm. In [29], mathematical forms are extracted to prove that the Gaussian kernel is the only transformation kernel that realizes scale transformation in different ways. The Gaussian filter is an optimal filter for establishing a linear scale space. For a 2D image *I*(*x*, *y*), the image filtered by a Gaussian filter can be represented as follows,

(5) L(x,y,δ)=G(x,y,δ)∗I(x,y)

where * represents the convolution operator. (x,y) represents the position of the pixels in the image, and G(x,y,δ) represents a Gaussian function whose formula can be expressed as

(6) $$G\left(x,y,\delta \right)=\frac{1}{2\pi \delta^{2}}e^{-\frac{{\left(x_{0}-m/2\right)}^{2}+{\left(y_{0}-n/2\right)}^{2}}{2\delta^{2}}}$$

where $\left(x_{0},y_{0}\right)$ is the coordinate of the center point. δ is a scale parameter, which is a continuous transformation value that determines the degree of smoothness of the transformed image. The larger δ is, the better the smoothness becomes, and the overall structure of the image can be captured. The smaller δ is, the weaker the smoothness becomes, and the details of the image can be retained. The algorithm here is based on Gaussian-scale space. Therefore, when scale transformation is performed, an operator is added so that the edge information can be preserved while extracting spatial features. The new scale transformation formula is

(7) $$L_{z}\left(x,y,\delta \right)=e^{\frac{1}{1+\nabla x_{i}}}\left(G\left(x,y,d\right)*I\left(x,y\right)\right)$$

where $\nabla x_{i}$ represents the Sobel operator, which can be expressed as

(8) $$\nabla x_{i}=\frac{1}{4}\sqrt{{\left(\nabla^{h}X_{i}\right)}^{2}+{\left(\nabla^{v}X_{i}\right)}^{2}+{\left(\nabla^{r}X_{i}\right)}^{2}+{\left(\nabla^{i}X_{i}\right)}^{2}}$$

where $\nabla^{h}X_{i}$, $\nabla^{v}X_{i}$, $\nabla^{r}X_{i}$, and $\nabla^{l}X_{i}$ denote the first-order horizontal, vertical, and two diagonal gradients of a pixel, respectively.

### 3.2. Nonlinear Multi-Scale Spatial Features

In order to obtain translation invariance, a non-subsampled contourlet (NSCT) is considered as a nonlinear multiscale spatial feature. Compared with two-dimensional discrete wavelet transform, contour wave transform is better at representing two-dimensional signals and can significantly improve the performance of image denoising, image texture features, and shape features extraction. Through the pyramid directional filter bank (pdfb), the image is decomposed into directional sub-bands of different scales by a contourlet. Pdfb consists of two parts: a Laplacian pyramid (LP) and a directional filter bank.

**Figure 1:**
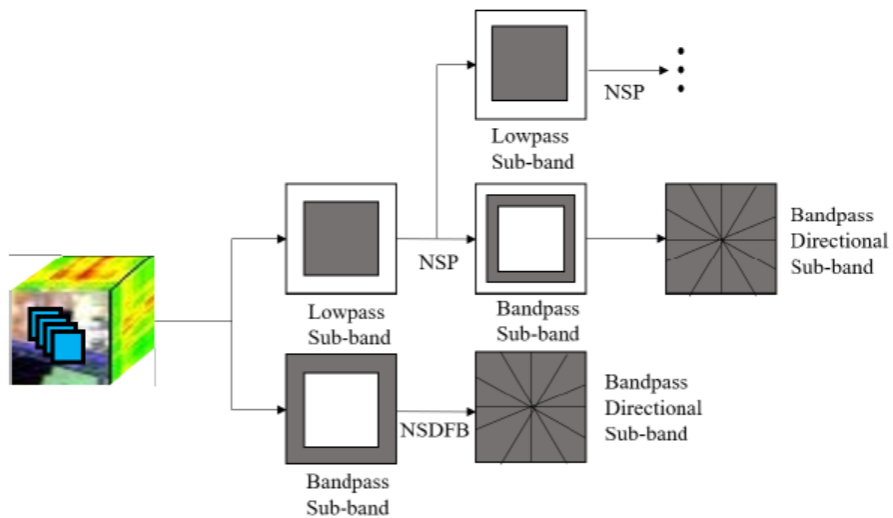
Illustration of the non-subsampled contourlet (NSCT).

Figure 1 shows the decomposition steps of NSCT. The basis of a NSCT is a non-subsampled pyramid (NSP) and non-subsampled directional filter banks (NSDFBs). There are two main steps in a NSCT: First, the input image is decomposed into high-pass and low-pass parts by an NSP tower, and second the high-frequency sub-band is then decomposed into multiple directional sub-bands by an NSDFB.

### 3.3. mGBDT

In this paper, after data processing and feature extraction of the hyperspectral images, the extracted features are used as input to the mGBDT model to train it. The mGBDT model uses the GBDT tree structure as the basic module, the network structure is simple, and the number of participants is small, so the training is convenient. For the adjustment of parameters, for different data, the deep network needs to adjust the network structure. The mGBDT model, with the same parameter settings, can meet the training needs of different data. The design of the model is ideal for theoretical analysis and practical training. Only simple hardware conditions are required to train the model. In order to improve classification accuracy, users must adjust a deep model. As the model is a multi-layered tree structure, backpropagation cannot be used during training to modify the parameters, like traditional deep neural networks. We know that, in a multilayer network, there is a forward propagation, *F*, between layers, i.e.,

(9) $$F^{t}\left(O_{i-1}\right)=O_{i}$$

The parameters of forward propagation are optimized to minimize the loss of the output of the last layer and groundtruth. Generally, it is effective to use backpropagation directly in different deep neural network models, but for multi-layer networks with a tree structure, it is necessary to define a parameter iteration mechanism. The mGBDT uses a pseudo-label idea to iterate through the parameters layer by layer. For the forward propagation of each layer, we set a reverse mapping,

(10) $$G_{i}^{t}\left(F_{i}^{t-1}\left(O_{i-1}\right)\right)\approx O_{i-1},$$

compute the loss and *G*,

(11) $${\hat{G}}^{t}=\mathrm{argmin}E_{x}\left[∥G_{i}\left(F_{i}\left(O_{i-1}+\varepsilon \right)-\left(O_{i-1}+\varepsilon \right)\right)∥\right],$$

and introduce some noise to increase model robustness. The purpose of the iterative update of *F* and *G* is to obtain the error between the ideal output and the actual output of the current layer. First, it is necessary to obtain the ideal output of each layer. This seems to be difficult, but a pseudo-label can be set to approximate the real output. The top-level pseudo-labels can be calculated using ground truth, i.e.,

(12) $$Z_{m}^{t}=OM-\partial \frac{\partial L\left(O_{M},y\right)}{\partial O_{M}}$$

Inverse mapping is used to update *Z* from back to front, and *F* can then be updated toward the residual:

(13) $$-\frac{\partial L\left(F_{M}^{t-1}\left(O_{M-1}\right),Z_{M}^{t}\right)}{\partial F_{M}^{t-1}\left(O_{M}-1\right)}$$

### 3.4. Hsi Classification Based on Feature Fusion and mGBDT

The selection of features is a key step in the hyperspectral classification task. This paper uses three features: EMP, linear multiscale features, and nonlinear multiscale features. EMP effectively extracts the morphological features of each layer of the hyperspectral image and retains the geometric information of the image well. Gaussian filtering and Sobel operators are used for linear multi-scale spatial features to sufficiently suppress image noise while retaining image edge features. Nonlinear multi-scale features use the NSCT feature, which has good multi-directionality and multi-scale properties, and have translation invariance, which can effectively extract the contour features of the image. The combination of linear and nonlinear features is effective. The edge and contour information of the image is preserved. The combination of the three features guarantees the adequacy, efficiency, and accuracy of the proposed features and provides favorable input features for subsequent classification models.

**Figure 2:**
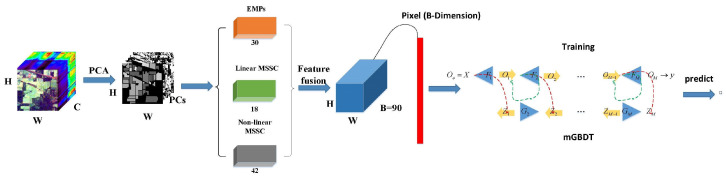
The overall framework of the proposed model.

In this paper, the proposed FF-DT is based on feature fusion and mGBDT. Figure 2 shows the architecture of the proposed method. Let $I\in R^{\;W*H*C}$ represent a hyperspectral image, and let $X^{n}\in R^{1*C}$, n∈[1,N] represent an HSI pixel. First, because the hyperspectral image has a high spectral dimension, we use the PCA method to reduce the redundancy, retaining the first principal components to contain most of the information $I_{pc}\in R^{\;W*H*C}$. The spectral-spatial features of the input data are then extracted by EMP, linear multi-scale spatial characteristics, and nonlinear multi-scale spatial characteristics. At last, each pixel from the hyperspectral images is transformed into a one-dimensional feature vector, $I\in R^{\;W*H*C}$.

On the basis of feature fusion, we introduce a multi-level gradient boosted decision tree (mGBDT). The mGBDT is a model based on a decision tree whose overall amount of parameters is small. Compared with many current deep models with wider and deeper network parameters, network parameter training is easier and faster, and it can obtain better results in a shorter time when the CPU and other hardware conditions are poor. In addition, in order to fully train a deep model with a large number of parameters, a sufficient amount of training data is required to improve model performance. However, the mGBDT can obtain better results with a small number of training samples. In this paper, we define the framework: input-l1-l2-output, which is simple but useful. More detailed parameter analysis will be discussed in the experiment section.

## 4. Experiment Designs

### 4.1. Datasets

In the experimental part, we use multiple datasets to verify the validity of the model. The images in the Pavia University dataset cover the city of Pavia in Italy. This dataset was captured by a reflective optics system imaging spectrometer (ROSIS) over the city. Each image is 610 * 340 pixels. The number of spectral bands is 103, with a spectral coverage ranging from 0.43 to 0.86 m and a spatial resolution of 1.3 m per pixel. There are nine different classes: asphalt, meadows, gravel, trees, painted metal sheets, bare soil, bitumen, self-blocking bricks, and shadows. Different colors denote different types.

The Indian Pines dataset depicts Indian pine forests in Indiana, Northwestern United States. They were captured by an Airborne Visible/Infrared Imaging Spectrometer (AVIRIS) sensor over Northwestern Indiana and contain 220 spectral bands with a spectral coverage ranging from 0.4 to 2.5 m. When there were 20 spectral bands that could not be reflected by water, they were removed. The remaining 200 spectral bands were chosen as the experimental objects, which include 16 classes.

The images in the Salinas dataset were captured by an AVIRIS sensor over the city of Salinas Valley in the state of California, USA. These images contain 224 bands with 16 classes, including vegetables, vineyard fields, and bare soil.

### 4.2. Parameter Analysis

The proposed method is based on extended morphological features and an mGBDT. First, at the data level, data preprocessing is required. For the redundant spectral dimensions of the original input data, PCA was used to extract principal components and reduce the data dimensions. The first five principal components in the spectral dimension were retained because they contain more than 99 percent of the information. EMP was then used to extract the morphological features of the hyperspectral data, with four openings and closings (range from 2 to 8, with a step size of 2). Each pixel was a 1-d vector with 90 dimensional features, and the model was trained and predicted using the mGBDT.

Furthermore, the maximum depth of each tree used by the forward network and inverse mapping in the model was 7, the learning rate was 0.2, one-hot coding training and prediction data was used, and the inverse mapping layer used Gaussian noise with a mean value of 0 and a standard deviation value of 0.3. In order to avoid training the inverse mapping at the top level, the top-level classification layer was set to a linear function with cross entropy loss, the learning rate was set at 0.03, and other layers used GBDT for inverse mapping.

## 5. Results

The proposed model was compared with SVM-RBF [30], SVM-EMP [31], CNN [32], 3D-CNN [33], FuNet-C [34], and MDGCN [35] to explore whether the proposed model was effective. SVM-RBF used the spectral features of the hyperspectral data as input for training. SVM-EMP used the EMP features as input for training. The CNN model used only spectral features and used a one-dimensional convolution kernel to extract them. 3D-CNN introduced the spatial dimension. The image blocks were input into the CNN network, and the results were output. FuNet-C is a semisupervised network based on graph neural network. MDGCN is applicable to the irregular image regions represented by graph topological information. This experiment used three hyperspectral datasets: the Pavia University, Indian Pines, and Salinas datasets.

### 5.1. Classification Results of the Pavia University Dataset

The Pavia University data were divided into a training set and a test set at a ratio of 5% and 95%. Each model used the same dataset to train and test.

**Figure 3:**
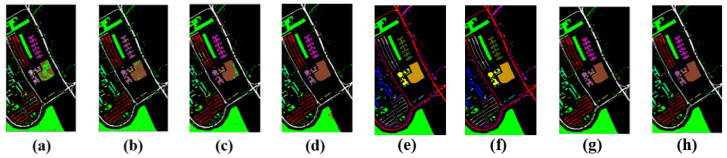
Classification results map of various algorithms on the Pavia University dataset: (**a**) RBF-SVM, (**b**) EMP-SVM, (**c**) 2D-CNN, (**d**) 3D-CNN, (**e**) FuNet-C, (**f**) MDGCN, (**g**) FF-DT, and (**h**) ground truth.

Table 1 and Table 2 and Figure 3 show the classification performances and classification visualizations of the Pavia University dataset for various algorithms. Figure 3 shows the results of seven different classification algorithms. Different colors represent different types of experimental objects. The many different colors between RBF-SVM (a) and ground truth (h) in the center of these two pictures indicate the poor classification performance of RBF-SVM (a). The EMP-SVM (b) outperforms the RBF-SVM (a), but there is a gap between EMP-SVM (b) and ground truth (h). The 2D-CNN (c) and 3D-CNN (d) obtain better classification results compared to the former two algorithms because of the complex computing models and hyperparameters. The 3D-CNN outperforms the 2D-CNN. The FuNet-C (e) and MDGCN (f), that are based on the graph, do not obtain a better performance than the mGBDT. Our proposed model FF-DT (g) performs the best in all the models, as there are significant similarities between the results of FF-DT (g) and ground truth (h). Furthermore, it relies on a small complex network and minimal computing conditions, making the HSI classification more practical.

Table 1 and Table 2 show that, for the dataset of Pavia University, the overall accuracy (OA) of SVM using EMP is greatly improved compared with that of SVM-PCA using spectral features. The CNN method effectively utilized the features of the hyperspectral images. Even the spectral dimension features are relatively good. The 3D-CNN extract spectral-spacial features of hyperspectral images were also improved. However, due to the structural limitations of CNNs, if a high precision is required, a large number of training data and a wider and deeper network are needed. The FuNet-C model is based on a graph requiring a high computation complexity; however, its classification accuracy on this dataset is lower than our proposed model. The same as MDGCN, that is based on graph neural network. The MDGCN performs worse than the mGBDT. The mGBDT network using EMP features can effectively and quickly train the network. In this experiment, the proposed model is 11% higher in OA than the r-SVM. Furthermore, it is 8.7% higher in KAPPA than the E-SVM. It can be seen that in most of types, FF-DT performs the best in all the algorithms. Even compared to the CNN based model, it obtains a great result in both training speed and classification accuracy in most of the class. It is shown in Table 2 that our proposed model obtains the best classification result with or without considering the weight of each component, illustrating the superiority of FF-DT.

### 5.2. Classification Results on the Indian Pines Dataset

The images in the Indian pines dataset have a small space size but contain 16 types of features. The data were divided into a training set and a test set at a ratio of 10% and 90%.

**Figure 4:**
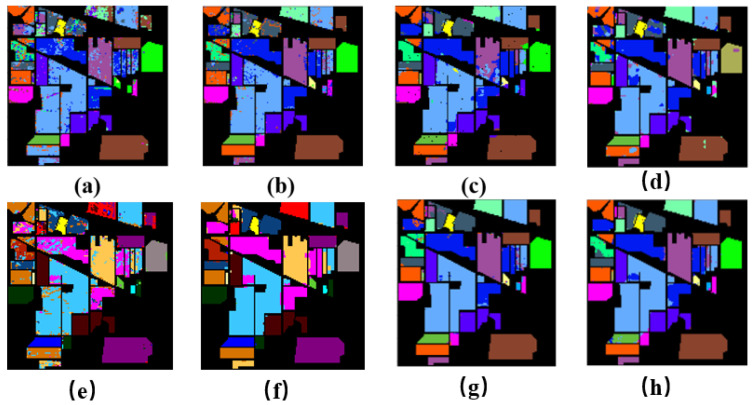
Classification results map of various algorithms on the Indian Pines dataset: (**a**) RBF-SVM, (**b**) EMP-SVM, (**c**) 2D-CNN, (**d**) 3D-CNN, (**e**) FuNet-C, (**f**) MDGCN, (**g**) FF-DT, and (**h**) ground truth.

The classification performance and visualization of the Indian Pine dataset by this experiment and multiple comparison algorithms are shown in Table 3 and Table 4 and Figure 4.

Figure 4 shows results of five different classification algorithms on the Indian Pines dataset. There are many noisy points in the RBF-SVM (a) and EMP-SVM (b), which indicates their poor classification performance. The 2D-CNN (c) and 3D CNN (d) outperform the former two algorithms, because their inner structures are complex. The FuNet-C (e) and MDGCN (f) obtain a better performance than the former models, for the complex network architecture based on graph. Our proposed model, FF-DT (g), is the most similar to the ground truth (h).

Table 3 and Table 4 show that the SVM classification and CNN effect are not particularly good if only spectral features are used. As the Indian Pines dataset is small, there are too many categories. However, extended morphological features can effectively represent the information contained in the hyperspectral images, and the performance of SVM is effectively improved. 3D-CNN used spectral-spacial information, and the classification effect was better. The FuNet-C and MDGCN perform well in this dataset; however, their classification accuracy are lower than our proposed mGBDT. With the importing of mGBDT algorithm. The fusion of the three features fully embodies the spatial and spectral features of the hyperspectral images and the spectral information of the datasets. Moreover, the mGBDT model makes a great contribution to the classification results. From the Table 3, the FF-DT model is 27% higher in AA than the r-SVM. In OA and KAPPA, it outperforms all the baselines. From Table 4, it is shown that the FF-DT obtains a best classification precision in 5% Indian Pines dataset. In weighted average value, FF-DT obtains the best performance in all the evaluation metrics, illustrating the importance of weighting each component in classification process.

### 5.3. Classification Results on the Salinas Dataset

The Salinas dataset was divided: 5% for training data and 95% for test data. Table 5 and Table 6 and Figure 4 show the classification results and classification visualizations of the different methods for the Salinas dataset.

**Figure 5:**
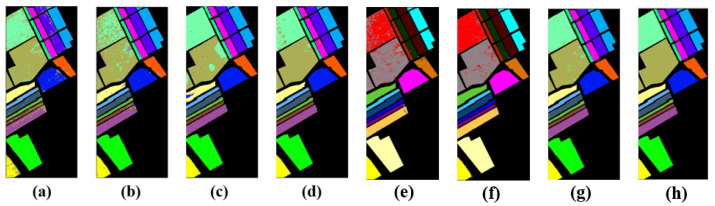
Classification results map of various algorithms on the Salinas dataset: (**a**) RBF-SVM, (**b**) EMP-SVM, (**c**) 2D-CNN, (**d**) 3D-CNN, (**e**) FuNet-C,(**f**) MDGCN, (**g**) FF-DT, and (**h**) ground truth.

Figure 5 shows the results of five different classification algorithms on the Salinas dataset. It can be seen that the SVM using EMP (b) features outperforms the RBF-SVM (a). Many different color points are shown in the RBF-SVM (a) and EMP-SVM (b), indicating that these two algorithms do not classify well. The 2D-CNN (c) and 3D CNN (d) outperform the former two algorithms, because they have imported spatial information and possess large amounts of hyperparameters. The FuNet-C model (e) and MDGCN model (f) conducted on the dataset show a good classification result, based on the graph neural network. Our proposed model, FF-DT (g), appears to have the least points compared to the other algorithms, representing its superiority in HSI classification.

Table 5 and Table 6 show that, due to the characteristics of the Salinas hyperspectral imagery, even if a support vector machine is used to train the spectral dimension, it can achieve better results. Similarly, a SVM using EMP features will have better results. Because of the integration of spatial dimensions, the 3D-CNN leads to an improved prediction accuracy than the 2D-CNN network. Furthermore, FuNet-C and MDGCN show much improvement in classification accuracy. However, the mGBDT network using feature fusion is still the best on the whole. From the Table 5, it can be seen that the FF-DT model is 21% higher in KAPPA than the r-SVM. In OA and AA, FF-DT obtains the best performance. From Table 6, we found that the FF-DT performs best in 5% Indian Pines dataset, especially when the weight of each component is considered. It also can be seen that the EMP-SVM performs great in all evaluation metrics, illustrating the superiority of extended morphological features.

### 5.4. The Effect of Multi-Feature Fusion

In order to show the effect of multi-feature fusion, we added a comparison experiment of single feature and multi-feature fusion, shown in Table 7. The experiment showed that multi-feature fusion has an improved classification effect. The feature vector dimensions extracted by EMP, Linear MSSC, Nonlinear MSSC, and Feature Fusion are 30, 18, 42, and 90, respectively. For the Pavia University dataset in Table 1, Feature Fusion was 9.1% higher than the single EMP feature, 4.6% higher than the single Linear MSSC, and 0.2% higher than the single Nonlinear MSSC. For the Salinas dataset in Table 1, Feature Fusion was 0.4% higher than the single EMP feature, 15.2% higher than the single Linear MSSC, and 0.8% higher than the single Nonlinear MSSC. The Indian Pines dataset had similar results compared to the Salinas dataset.

In the experiments on the hyperspectral data, we used the same model structure, input-32-32-output, for the three different datasets with different numbers and classifications. The classification results show that the mGBDT model is more robust. In terms of training time, the mGBDT model was trained on a personal PC. The time required was also close to the time required to run a deep network on a GPU device.

**Figure 6:**
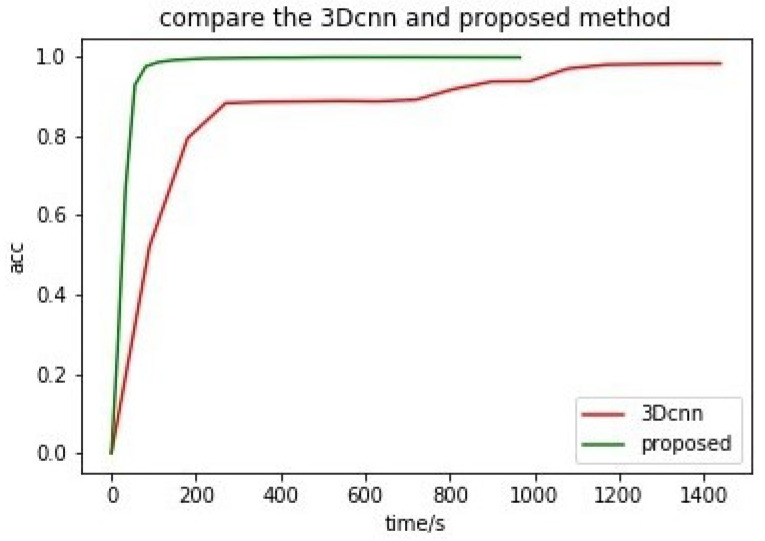
Speed comparison of 3D-CNN and the proposed FF-DT.

Our proposed method consists of feature fusion and an effective classifier mGBDT. In order to obtain better classification accuracy, we need to spend a lot of effort to adjust the parameters of deep model, and when data changes, the deep network also needs to adjust the network structure. The mGBDT is a new model of tree structure proposed in 2018. Compared with the deep learning model, the design of the model is easy for theoretical analysis and practical training, and only requires simple hardware conditions to perform model training. Considering the advantages of mGBDT, we first introduce mGBDT to hyperspectral classification. The mGBDT model, with the same parameter settings, can meet the training of different data (We use the same parameter settings, in the experiment of hyperspectral data. The model structure, intput-32-32-output, can obtain better classification results, showing that the mGBDT model has better robustness.), and mGBDT also has other advantages. To illustrate the advantages of mGBDT in terms of computational consumption, we have added speed comparison experiments of 3D-CNN and the proposed FF-DT shown as Figure 6. Graphics processing often requires a higher-cost GPU, while mGDBT requires only a normal CPU to achieve faster convergence speed. (The selected data set is the commonly used hyperspectral data set Pavia, 3D-CNN uses GPU: GeFore GTX 1080Ti; the proposed FF-DT uses CPU: Intel i7). Compared with 3D-CNN, shorter training time and faster convergence is much more competitive in some certain scenarios. Therefore, this model can be effectively applied to some lightweight remote sensing devices.

In conclusion, the FF-DT method is proposed to solve the problem that the traditional method cannot effectively use the spatial information of hyperspectral data. The extracted EMP features greatly preserve the geometric information of the image, while the linear and nonlinear multi-scale spatial features well retain the edge and contour information of the image. After the three features are fused, the target image’s effective information will be well retained, which could be beneficial for subsequent classification tasks. On the classifier, we choose the mGBDT with strong representation ability and adaptive adjustment ability of structure complexity to improve the classification performance. From the comparative analysis with three comparative experiments, it can be seen that the algorithm in this chapter excels in the classification task of hyperspectral images. Even for Indian Pines with small homogeneous regions, the algorithm in this chapter can greatly retain its edge information, making the classification process more efficient and accurate. From the experimental classification accuracy table and classification result graph, the efficiency of the algorithm in this chapter can be seen.

## 6. Conclusions

Models based on deep convolutional neural networks are widely used in hyperspectral image processing fields, such as image classification, but deep networks require sufficient training data, appropriate adjustments of many parameters, and high hardware conditions. It is difficult to obtain good results for small sample data because of the disadvantages of back-propagation algorithms. To overcome these shortcomings, the proposed method in this paper introduces feature fusion and an mGBDT model to better extract and classify the spectral and spatial features of hyperspectral images. First, principal component analysis was used to reduce the dimensions of the data. Extended morphology profiles (EMPs), linear multi-scale spatial characteristics, and nonlinear multi-scale spatial characteristics were used to extract spectral features. Finally, a mGBDT model was constructed for classification. The model based on the decision tree requires fewer hyperparameters and simple training conditions. This paper proposes an algorithm that can achieve relatively good classification accuracy with limited hardware conditions and short iterations. In the three hyperspectral image classification experiments, the proposed model combined the excellent performance of the tree and the use of EMP features to effectively extract the spatial and spectral information of hyperspectral images and can thus achieve better performance than other current classical models.

## Figures and Tables

**Table 1:** Classification results of the Pavia University dataset.

Class	r-SVM	E-SVM	CNN	3D-CNN	FuNet-C	MDGCN	FF-DT
1	0.8790	0.9910	0.9960	0.9860	0.9492	0.9896	0.9980
2	0.8830	0.8770	0.9840	0.9720	0.9917	0.9963	0.9990
3	0.7090	0.9980	0.8310	0.9710	1.0000	0.8976	0.966
4	0.9470	0.9990	0.8360	0.9820	0.9782	0.9509	0.9930
5	0.9990	1.0000	0.9780	1.0000	1.0000	0.9728	1.0000
6	0.8680	0.9740	0.9150	1.0000	0.9990	0.9740	1.0000
7	0.8340	0.9960	0.9870	0.9980	0.8592	0.9804	0.9980
8	0.8390	0.9940	0.9360	0.9980	0.9025	0.9635	0.9980
9	1.0000	1.0000	0.8820	0.8910	0.9993	0.9039	0.9840
OA	0.8780	0.9340	0.9530	0.9810	0.9720	0.9881	0.9980
AA	0.8840	0.9810	0.9270	0.9710	0.9591	0.9758	0.9970
KAPPA	0.8340	0.9110	0.9380	0.9720	0.9629	0.9841	0.9980

**Table 2:** 5% Pavia University dataset.

	Macro Avg			Weighted Avg		
	Precision	Recall	F1-Score	Precision	Recall	F1-Score
EMP-SVM	98.1%	98.4%	98.2%	98.8%	98.8%	98.7%
RBF-SVM	98.1%	97.1%	97.6%	98.5%	98.4%	98.4%
CNN	98.2%	97.7%	97.9%	98.6%	98.6%	98.5%
3D-CNN	95.2%	93.8%	94.3%	95.0%	94.8%	94.7%
FF-DT	99.8%	99.7%	99.7%	99.8%	99.8%	99.8%

**Table 3:** Classification results of the Indian Pines dataset.

Class	r-SVM	E-SVM	CNN	3D-CNN	FuNet-C	MDGCN	FF-DT
1	0.1432	0.9296	0.9723	0.8053	0.8793	0.8857	0.7863
2	0.6663	0.8839	0.8732	0.9042	0.7672	0.9275	0.9286
3	0.6053	0.8924	0.9113	0.8796	0.8256	0.9434	0.9943
4	0.5474	0.7662	0.8591	0.6023	0.7394	0.9553	0.9766
5	0.8537	0.8535	0.6940	0.8931	0.9271	0.9352	0.8323
6	0.9747	0.9713	0.9667	0.9740	0.9735	0.9803	1.0000
7	0	0.5772	0.5216	0.9129	0.9590	0.8176	1.0000
8	0.9957	1.0000	1.0000	0.9645	0.9841	0.9939	0.9769
9	0.1677	0.5561	0.4528	0.8236	1.0000	0.8058	0.9843
10	0.7377	0.8837	0.8346	0.963	0.7947	0.8997	0.7714
11	0.8566	0.9128	0.9377	0.949	0.8767	0.9776	0.9798
12	0.6223	0.8242	0.8768	0.7524	0.7641	0.9417	0.9890
13	0.9952	0.9958	0.9249	0.9125	0.9936	0.9824	0.8083
14	0.9693	0.9972	0.9764	0.9846	0.9433	0.9811	0.9981
15	0.464	0.9224	0.9595	1.000	0.6738	0.9555	0.7786
16	0.9171	0.8811	0.4562	0.9632	0.9512	0.8175	0.9711
OA	0.7875	0.9113	0.9123	0.9256	0.8797	0.9650	0.9721
AA	0.6572	0.8674	0.8264	0.8422	0.9033	0.9493	0.9233
KAPPA	0.7557	0.8981	0.8933	0.9142	0.8629	0.8601	0.9662

**Table 4:** 5% Indian Pines dataset.

	Macro Avg			Weighted Avg		
	Precision	Recall	F1-Score	Precision	Recall	F1-Score
EMP-SVM	94.9%	96.2%	95.4%	93.6%	93.5%	93.5%
RBF-SVM	84.9%	81.4%	82.4%	96.0%	96.4%	96.1%
CNN	92.1%	92.5%	92.0%	96.2%	96.3%	96.1%
3D-CNN	91.3%	92.6%	91.7%	91.4%	91.3%	91.2%
FF-DT	96.0%	92.3%	93.9%	97.1%	97.0%	97.0%

**Table 5:** Classification results of the Salinas dataset.

Class	r-SVM	E-SVM	CNN	3D-CNN	FuNet-C	MDGCN	FF-DT
1	0.9991	1.0000	0.9780	0.9860	0.9951	0.9711	1.0000
2	0.9911	1.0000	1.0000	1.0000	0.9985	0.8983	0.9998
3	0.9642	0.9983	1.0000	1.0000	0.9690	0.9879	0.9570
4	0.9863	0.9966	0.9777	0.9538	0.9905	0.9741	0.9988
5	0.9954	0.9993	1.0000	0.9670	0.9569	0.9824	0.8834
6	1.0000	1.0000	0.9941	1.0000	0.9990	0.9947	0.9866
7	0.9994	1.0000	0.9972	1.0000	0.9983	0.9971	0.9957
8	0.7453	0.7369	0.8694	0.9826	0.8655	0.8490	1.0000
9	0.9914	0.9975	1.0000	0.9987	0.9817	0.9878	0.9985
10	0.8313	1.0000	0.9816	0.9954	0.9676	0.9853	0.9797
11	0.9414	1.0000	0.9855	1.0000	0.9635	0.9916	0.9994
12	0.9715	1.0000	0.9999	0.9728	1.0000	0.9957	1.0000
13	0.9493	1.0000	1.0000	0.9983	0.9975	0.9944	1.0000
14	0.9795	0.9997	0.9914	0.9947	0.9473	0.9944	0.9990
15	0.8013	0.9416	0.9993	0.9193	0.7846	0.9616	1.0000
16	0.9985	1.0000	0.9892	0.9975	0.9886	0.9771	0.9993
OA	0.9023	0.9225	0.9736	0.9813	0.9422	0.9564	0.9956
AA	0.9465	0.9795	0.9855	0.9858	0.9686	0.9801	0.9870
KAPPA	0.8883	0.9114	0.9726	0.9785	0.9356	0.9515	0.9941

**Table 6:** 5% Salinas dataset.

	Macro Avg			Weighted Avg		
	Precision	Recall	F1-Score	Precision	Recall	F1-Score
EMP-SVM	98.8%	98.5%	98.6%	97.1%	97.1%	97.1%
RBF-SVM	98.4%	98.7%	98.5%	96.7%	96.7%	96.7%
CNN	98.7%	98.8%	98.7%	98.4%	98.9%	98.5%
3D-CNN	94.9%	94.3%	94.0%	90.8%	89.8%	88.9%
FF-DT	99.4%	98.7%	99.0%	99.5%	99.5%	99.5%

**Table 7:** Classification accuracy (OA) of single and fusion features.

Features	Pavia U	Salinas	Indian Pines
EMP (30)	0.9074	0.9916	0.9689
Linear MSSC	0.9522	0.8433	0.8510
Non-Linear MSSC	0.9961	0.9876	0.9675
Feature Fusion	0.9984	0.9955	0.9707

## Data Availability

Data available in a publicly accessible repository The data presented in this study are openly available in https://dx.doi.org/10.21227/eqk7-wa46.
